# MPC1, a key gene in cancer metabolism, is regulated by COUPTFII in human prostate cancer

**DOI:** 10.18632/oncotarget.7405

**Published:** 2016-02-15

**Authors:** Leiming Wang, Mafei Xu, Jun Qin, Shih-Chieh Lin, Hui-Ju Lee, Sophia Y. Tsai, Ming-Jer Tsai

**Affiliations:** ^1^ Department of Molecular and Cellular Biology, Baylor College of Medicine, Houston, Texas 77030, USA; ^2^ Institute of Health Sciences, Shanghai Institutes for Biological Sciences, Chinese Academy of Sciences and Shanghai JiaoTong University School of Medicine, Shanghai 200031, China

**Keywords:** chicken ovalbumin upstream promoter-transcription factor II (COUP-TFII), mitochondrial pyruvate carrier 1 (MPC1), prostate cancer, glycolysis, tumor growth

## Abstract

Mitochondrial pyruvate carrier 1 (MPC1) and MPC 2 form a transporter complex in cells to control pyruvate transportation into mitochondria. Reduced expression of MPC1 disrupts the transporter function, induces metabolic shift to increase glycolysis, and thus plays important roles in several diseases, including cancer. However, the role of MPC1 in prostate cancer and the underlying mechanism causing the down-regulation of MPC1 in tumor cells remain to be defined. Here, we show that MPC1 serves as a critical regulator of glycolysis in prostate cancer cells, which in turn controls cancer cell growth, invasion, and the tumorigenic capability. More importantly, we identified that chicken ovalbumin upstream promoter-transcription factor II (COUP-TFII), a steroid receptor superfamily member, transcriptionally regulates the expression of MPC1. We further demonstrate that COUP-TFII, which is upregulated in the prostate cancer patient, regulates MPC1 and glycolysis to promote tumor growth and metastasis. Our findings reveal that COUP-TFII represses MPC1 expression in prostate cancer cells to facilitate a metabolism switch to increase glycolysis and promote cancer progression. This observation raises an intriguing possibility of targeting COUP-TFII to modulate cancer cell metabolism for prostate cancer intervention.

## INTRODUCTION

Cancer cells have a distinct metabolism profile as compared to normal cells. They tend to have a high rate of glycolysis [1]. This phenomenon, also known as the “Warburg effect”, is related to pyruvate metabolism change caused by up-regulated glycolytic genes and suppression of mitochondrial pyruvate transportation and reduction of pyruvate decomposition in mitochondria [2, 3]. The mitochondrial pyruvate carrier (MPC) genes, MPC1 and MPC2, were recently identified to form a transporter complex to control rate-limiting pyruvate transportation through the inner mitochondrial membrane [4, 5]. Deficiencies in MPC function block pyruvate entry into the TCA cycle, which leads to a metabolism switch to increase glycolysis and the compensatory usage of glutamine [6–8]. Thus, MPC function has been implicated to be important for multiple diseases, including cancer [9, 10]. Interestingly, down-regulation of MPC1 has been shown in many cancers, and co-expression of MPC1 and MPC2 inhibits colon cancer cell growth [11]. However, the molecular mechanism underlying the down-regulation of MPC1 in diseases remains largely undefined.

Chicken Ovalbumin Upstream Promoter Transcription Factor II (COUP-TFII) is an orphan nuclear receptor that belongs to the steroid receptor superfamily [12]. COUP-TFII is broadly expressed in multiple tissues throughout embryonic development and is crucial for organogenesis, but its expression is greatly reduced in adult tissues, especially in epithelial cells where cancers normally arise from [13]. Recently, increasing evidence indicates that COUP-TFII is dysregulated in multiple cancer types and plays important roles in cancer development [14–17]. In prostate cancer, COUP-TFII collaborates with PTEN loss to promote cancer progression and metastasis [18]. However, the roles of COUP-TFII in prostate cancer metabolism have yet to be delineated.

Here, we show that COUP-TFII suppresses MPC1 expression to regulate cancer cell metabolism. Reducing COUP-TFII expression ameliorates prostate tumor growth. Our results raise the possibility that COUP-TFII could be a therapeutic target for treatment of prostate cancer.

## RESULTS

### MPC1 is down-regulated in prostate cancer specimens and overexpression of MPC1 suppresses tumor cell growth and invasion

**Figure 1:**
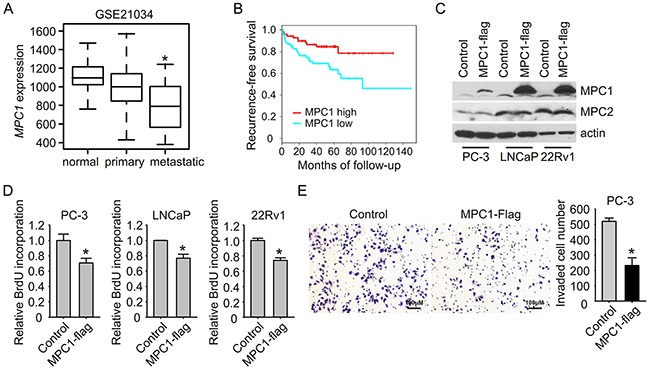
MPC1 suppresses prostate cancer cell growth and invasion **A.** MPC1 expression in human primary and metastatic prostate cancer samples and control normal adjacent benign prostate from patients in Taylor dataset GSE21034. *P<0.05; metastatic versus primary. **B.** Kaplan-Meier plot of survival rate based on MPC1 expression index in patients from Taylor dataset GSE21034. **C.** PC-3 cells with MPC1 overexpression were generated using lentivirus delivered MPC1-Flag. MPC1 overexpression was examined by immunoblotting. **D.** Cell growth was measured using BrdU incorporation assay, and normalized to control virus infected group. *P<0.05. **E.** Cell invasion was measured in PC-3 cells with or without MPC1 overexpression. Invaded cell number in the field was shown in the right panel. *P<0.05.

Increased glycolysis is a common hallmark of metabolism changes in cancer cells. To study glycolysis in prostate cancer, we used Oncomine expression analysis database to find the dysregulated glycolytic genes. Among the glycolytic genes, we found the down-regulation of MPC1 (BRP44L), and the up-regulation of HK2 and GPI in prostate cancer, which also showed similar dysregulation patterns in other types of cancers (Supplementary Figure S1). We chose MPC1 for further study because MPC1 is also down-regulated in prostate cancer tissues in other clinical datasets including GSE21034 [19] and TCGA (Figure 1A and Supplementary Figure S2). Interestingly, MPC1 is further down-regulated in metastatic prostate tumors in comparison to primary tumors (Figure 1A), indicating that MPC1 down-regulation may predict a more aggressive prostate cancer. More importantly, patients having low levels of MPC1 expression showed poor prognosis (Figure 1B).

To investigate the function of MPC1 in prostate cancer, we overexpressed MPC1 in prostate cancer cells using lentivirus delivered MPC1 with C terminal flag tag (Figure 1C). MPC1 protein levels were significantly increased after overexpression without affecting MPC2 protein expression. Consistent with the decreased MPC1 expression observed in patients, forced expression of MPC1 reduced cell growth of prostate cancer cells as indicated by BrdU incorporation (Figure 1D). To investigate whether MPC1 affects cancer invasion, we used a transwell chamber assay to measure the prostate cancer cell invasion ability. As shown in Figure 1E, we found that overexpression of MPC1 in PC-3 cells led to about 60% inhibition of cell invasion. This result of MPC1 dependent down regulation of invasive activity is consistent with the further down-regulation of MPC1 in metastatic prostate tumor tissue compared to primary tumor (Figure 1A), and suggests that MPC1 may also play important roles in prostate tumor aggressiveness. Taken together, these results indicate that MPC1 has a tumor suppressor function in prostate cancer cells.

### COUP-TFII suppresses MPC1 expression in prostate cancer

**Figure 2:**
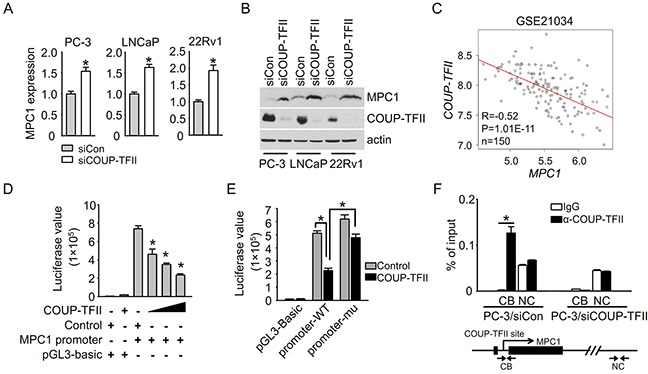
COUP-TFII suppresses MPC1 expression **A, B.** MPC1 mRNA and protein levels in PC-3, LNCaP and 22Rv1 cells were examined by q-PCR and immunoblotting respectively at 48 hours after siRNA transfection. *P<0.05. **C.** The correlation between COUP-TFII and MPC1 expression levels in prostate cancer samples from patients in Taylor dataset GSE21034. Patient number is 150. The X and Y axis values are log2 transformed signal values. **D.** Total amount of 1 μg indicated vectors were transfected into 293T cells per well of a 12-well plate. In each well, promoter vector was 0.33 μg, COUP-TFII vector was 0.165, 0.33 and 0.66 μg as increased. Control vector was added to get the equal amount in each well. Luciferase assay was performed 48 hours after transfection. *P<0.05; versus control and MPC1 promoter transfection group. MPC1 promoter is from −1194 to 85 region. **E.** MPC1 promoter (promoter-WT) or MPC1 promoter with mutated COUP-TFII binding site (promoter-mu) were transfected with COUP-TFII or control vector into 293T cells. Luciferase assay was performed 48 hours after transfection. *P<0.05. **F.** ChIP assay was performed at 48 hours after siRNA transfection. The pair of primers near COUP-TFII binding site (CB) and negative control pair of primers (NC) were used for q-PCR assay. *P<0.05.

To investigate how MPC1 is down-regulated in prostate cancer, we searched for the predicted transcriptional binding sites on the MPC1 promoter using PROMO TRANSFAC. Interestingly, there is a potential COUP-TFII binding site within the MPC1 promoter (−225 upstream). In addition, increased expression of COUP-TFII was observed in prostate cancer patients and it is further increased in metastatic patients, which is consistent with the possibility of negative regulation of MPC1 by COUP-TFII. Most importantly, an increased expression of MPC1 was also found in our earlier microarray analysis of COUP-TFII knockdown PC-3 cells (1.3 fold change in GSE33182). Further validation supported that COUP-TFII negatively regulates MPC1 expression, as we found that mRNA and protein levels of MPC1 were both increased subsequent to knock down of COUP-TFII expression in all three tested prostate epithelial cells, PC-3, LNCaP and CWR22Rv1 (Figure 2A and 2B). The negative transcription regulation was further validated by prostate cancer patient specimen analysis, in which there was a significant and negative correlation between MPC1 and COUP-TFII expression, consistent with the notion that COUP-TFII may serve as an upstream regulator to control MPC1 expression in patients (Figure 2C). In addition to prostate epithelial cells, we also found that MPC1 expression is elevated when COUP-TFII was knocked-down in other cell lines, including HeLa, HUVEC, MDA-MB-231, SK-HEP-1 and HepG2 (Supplementary Figure S3A and S3B). Taken together, these results indicate that regulation of MPC1 expression by COUP-TFII is not restricted to prostate cancer cells.

To dissect how COUP-TFII regulates MPC1 expression, we first carried out luciferase reporter assay using MPC1 promoter driven reporter constructs. The results showed that COUP-TFII repressed MPC1 promoter driven luciferase activity in a dose-dependent manner (Figure 2D), suggesting that COUP-TFII directly regulates MPC1 promoter transcriptional activity. To investigate whether COUP-TFII works through its binding site on the MPC1 promoter, we mutated the binding site and found that the inhibition of MPC1 promoter activity by COUP-TFII was significantly abrogated when the COUP-TFII binding site in the MPC1 promoter was mutated (Figure 2E). To further confirm that COUP-TFII binds to the site inside cells, we performed Chromatin Immunoprecipitation (ChIP) Assays (ChIP-qPCR). As shown in Figure 2F, COUP-TFII was recruited to the MPC1 promoter region containing the potential COUP-TFII binding site, but not to a control region lacking a COUP-TFII binding site. The binding was further validated in the COUP-TFII knocked-down PC-3 cells, in which a drastic reduction of COUP-TFII recruitment to the MPC1 promoter was observed (Figure 2F). Collectively, these results showed that COUP-TFII is recruited to the MPC1 promoter to suppress MPC1 expression.

### COUP-TFII regulates glycolysis in prostate cancer cells

**Figure 3:**
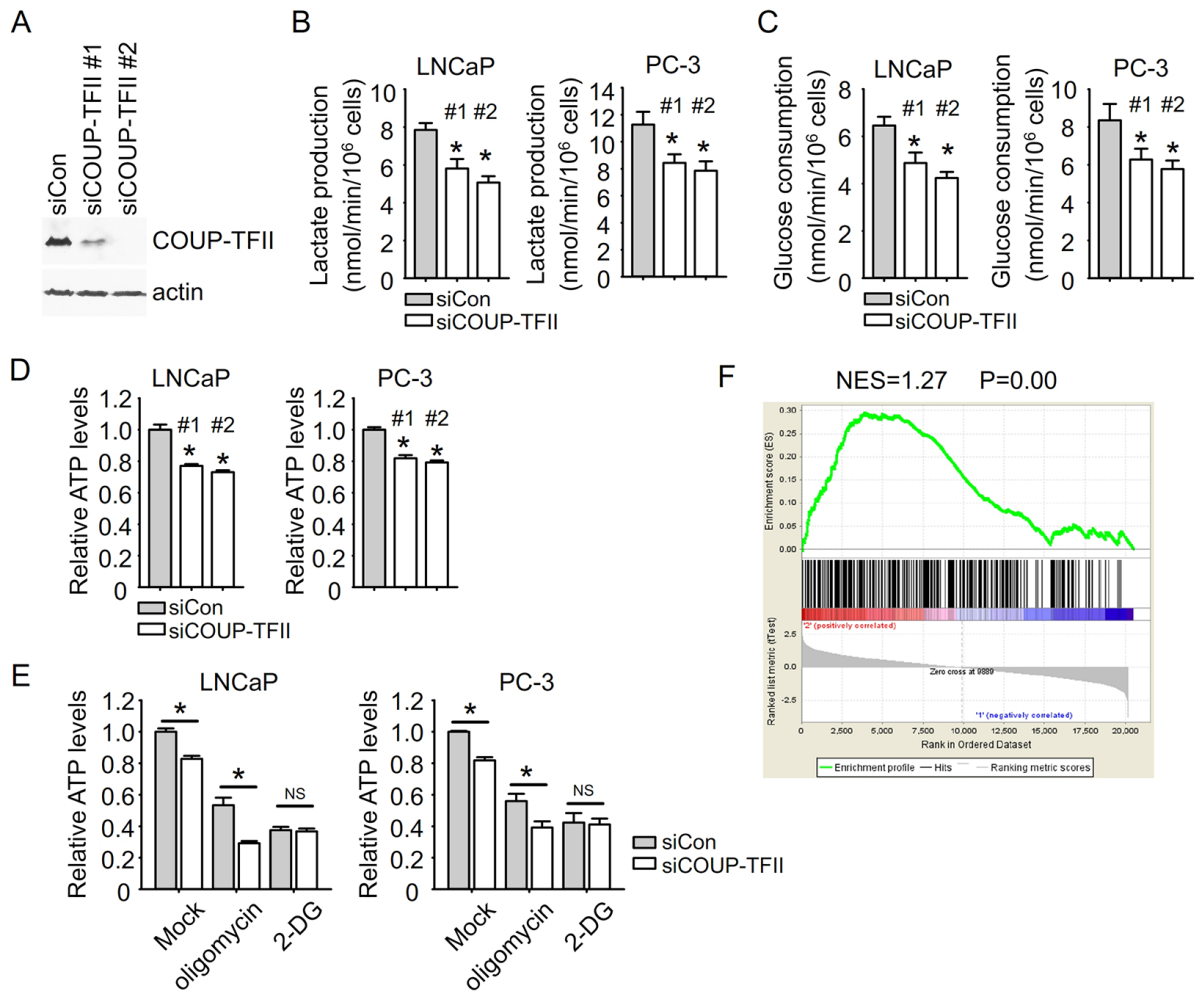
COUP-TFII regulates glycolysis in prostate cancer cells **A.** Two different siRNAs targeting COUP-TFII were transfected into PC-3 cells to measure their ability of inhibiting COUP-TFII expression. **B, C, D.** Cells were transfected with siRNAs. Fresh medium was exchanged 24 hours after siRNA transfection. After 24 hours, medium was collected for lactate and glucose assays, and cells were submitted to ATP assay. *P<0.05; versus siCon group. **E.** Cells were exchanged with fresh medium containing 10 μM oligomycin or 10 mM 2-DG at 24 hours after siRNA transfection. After 24 hours, cells were submitted to ATP assay. *P<0.05. NS; no significant difference. **F.** Gene Set Enrichment Analysis (GSEA) was carried out. COUP-TFII regulated genes in PC-3 cells (GSE33182) were ranked. Glycolysis regulated genes in 2-DG treated cancer cells (GSE16816) were used as the test set.

We further asked whether COUP-TFII could affect glycolysis in prostate cancer cells. Using two different siRNAs targeting COUP-TFII to suppress its expression (Figure 3A), we found that COUP-TFII knockdown significantly inhibited glycolysis as indicated by the reduction of lactate production and glucose consumption. The inhibition of glycolysis by COUP-TFII knockdown is observed in all of our tested prostate cancer cell lines, including LNCaP, PC3, CWR22Rv1, VCaP and LNCaP-abl (Figure 3B and 3C, Supplementary Figure S4A and S4B), implicating a broad impact of COUP-TFII on glycolysis of prostate cancer cells. Treatment of glycolysis inhibitor 2-DG greatly reduced glycolysis and abolished the COUP-TFII effect on glycolysis (Supplementary Figure S4C and S4D). These results indicate that suppressing COUP-TFII expression indeed leads to reduction of glycolysis.

Glycolysis and oxidative phosphorylation both produce ATP to support cellular energy expenditure. In cancer cells, elevated glycolysis plays a more prominent role in ATP supply than normal cells. Consistent with reduced glycolysis, we also found a reduction of levels of ATP upon knockdown of COUP-TFII in prostate cancer cells (Figure 3D). Using 2-DG and oligomycin to block glycolysis and oxidative phosphorylation generated ATP, respectively, we found that COUP-TFII knockdown was still able to reduce ATP levels in oligomycin treated cells, but not in 2-DG treated cells (Figure 3E), suggesting that COUP-TFII mainly regulates ATP generated by glycolysis in prostate cancer cells. Furthermore, the role of COUP-TFII in glycolysis was manifested by Gene Set Enrichment Analysis (GSEA). We found that the glycolysis regulated gene signature is significantly enriched in COUP-TFII regulated gene profiles, which corroborated the notion that COUP-TFII is important for glycolysis in prostate cancer cells (Figure 3F).

### MPC1 knockdown diminishes the effect of COUP-TFII on glycolysis

**Figure 4:**
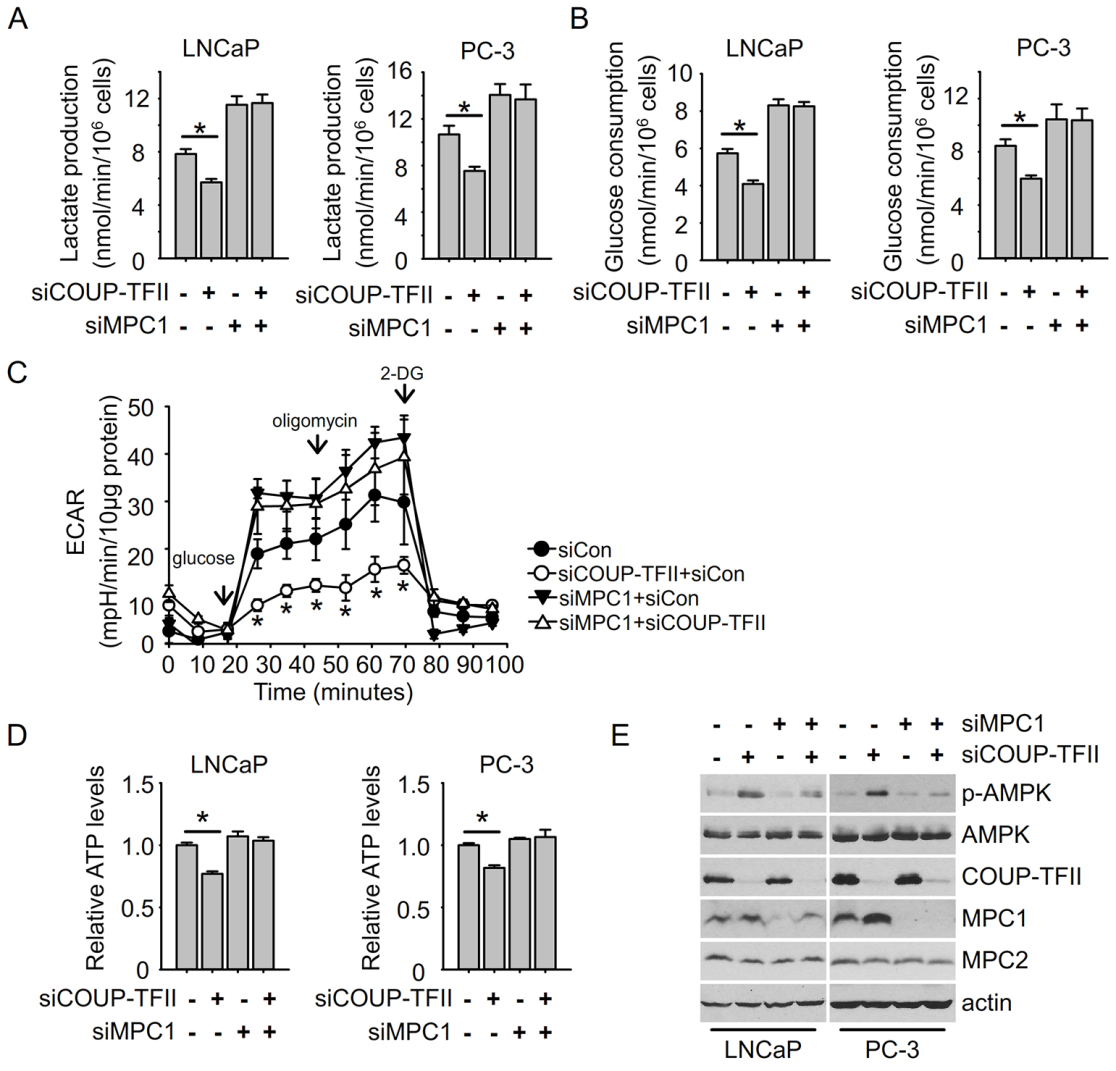
MPC1 knockdown diminishes the effect of COUP-TFII knockdown on inhibiting glycolysis **A, B.** Lactate production and glucose consumption were detected in cells 48 hours post-transfection with indicated siRNAs. *P<0.05. **C.** Glycolysis stress test was performed using seahorse XF24 instrument. Extracellular acidification rate (ECAR) was measured in indicated time points. *P<0.05, compared to corresponding time point of siCon group. **D.** ATP levels were detected in cells at 48 hours after transfection with indicated siRNAs. *P<0.05. **E.** Immunoblotting was performed to detect indicated protein levels in cells 48 hours post-transfection with indicated siRNAs.

Since COUP-TFII regulates MPC1 expression and MPC1 is important for glycolysis, we asked whether MPC1 mediates COUP-TFII regulation of glycolysis. As expected, MPC1 knockdown increased lactate production and glucose consumption in prostate cancer cells, hallmarks of increased glycolysis. More importantly, in the absence of MPC1, COUP-TFII failed to regulate glycolysis (Figure 4A and 4B). Through real-time measurement of the extracellular acidification rate (ECAR) of cells cultured in different conditions, a glycolysis stress test can assess the three key parameters of glycolytic function: glycolysis (glucose loaded condition), glycolytic capacity (oligomycin loaded condition to induce the maximal glycolysis) and glycolytic reserve (glycolytic capacity minus glycolysis). We found that COUP-TFII knockdown reduced glycolysis, glycolytic capacity and reserve, while MPC1 knockdown increased glycolysis levels and glycolytic capacity (Figure 4C). More importantly, the depletion of MPC1 abolished the reduction of glycolysis induced by COUP-TFII knockdown. Similarly, while knockdown of MPC1 had little effect on levels of ATP in either LNCaP or PC-3 cells, its depletion restored the levels of ATP in COUP-TFII knockdown cells to levels comparable to the controlled cells (Figure 4D). We further measured AMPK phosphorylation levels since reduction of ATP levels promotes AMPK activation. Consistent with changes in ATP levels, COUP-TFII knockdown led to an increase in AMPK phosphorylation, and this increase was abolished in the absence of MPC1 (Figure 4E). In the meantime, we noticed that MPC2 expression was not affected upon MPC1 knockdown. Collectively, these results indicate that MPC1 is essential for the COUP-TFII dependent regulation of glycolysis.

### MPC1 is important for COUP-TFII regulated prostate cancer cell growth and invasion

**Figure 5:**
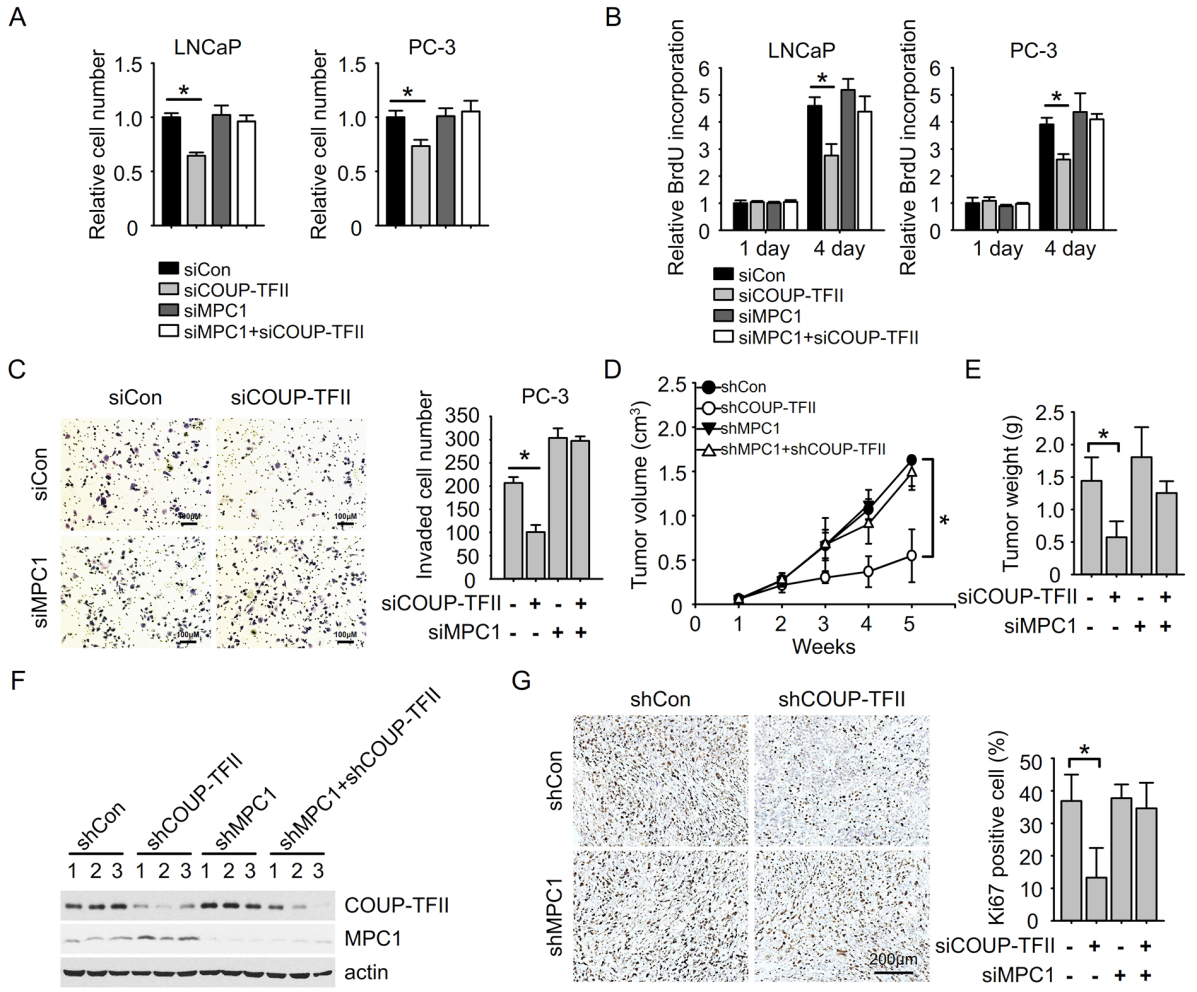
MPC1 knockdown diminishes the effect of COUP-TFII knockdown on inhibiting prostate cancer cell growth and invasion **A.** Cell number was counted 3 days after siRNA transfection. *P<0.05. **B.** BrdU incorporation assay was performed 3 days after siRNA transfection. *P<0.05. **C.** Cell invasion was measured 2 days after siRNA transfection. Invaded cell number in the field was shown in the right panel. *P<0.05. **D.** PC-3 cells with indicated stable shRNAs expression were subcutaneously injected into mice. The tumor size was measured 2 weeks later when palpable tumors were observed. *P<0.05. **E.** Removed tumor masses were weighted and quantified. *P<0.05. **F.** Tumor tissues were submitted to Immunoblotting for indicated proteins. **G.** Ki67 was stained in the tumor samples. The percentage of Ki67 positive cells in the fields was calculated and shown in the right panel. *P<0.05.

Next, we investigated the effect of COUP-TFII and MPC1 on prostate cancer progression. We showed previously that COUP-TFII knockdown reduced cell growth. Here, we found that MPC1 knockdown didn't apparently affect cell growth, but depletion of MPC1 largely nullified the effect on cell growth exerted by COUP-TFII (Figure 5A). Consistently, the reduction of BrdU incorporation upon COUP-TFII knockdown was also abrogated by MPC1 depletion (Figure 5B). In addition, the prostate cancer cell invasion was inhibited to about 50% upon COUP-TFII knockdown. In contrast, depletion of MPC1 increased cell invasion (Figure 5C). Most importantly, COUP-TFII knockdown was not able to inhibit cell invasion in the absence of MPC1. These results show that MPC1 is important for COUP-TFII regulated prostate cancer cell growth and invasion, and suggest that MPC1 works downstream of COUP-TFII in regulating glycolysis to control prostate cancer becoming aggressive and with a poor prognosis.

Finally, we carried out an *ex vivo* assay to ask whether COUP-TFII regulates tumor growth *in vivo* in a MPC1 dependent manner. First, we generated PC3 cells with stable COUP-TFII knockdown, MPC1 knockdown or double knockdown cells with shRNAs. These cells were then subcutaneously injected into SCID mice to induce prostate tumor formation (Figure 5D). With this assay, we showed that COUP-TFII knockdown inhibited tumor growth and tumor burden, and this inhibition was abolished when MPC1 expression was repressed (Figure 5D, and 5E, and Supplementary Figure S5), suggesting that MPC1 is critical for COUP-TFII regulated tumor growth. As expected, COUP-TFII knockdown induced the expression of MPC1 in tumor samples (Figure 5F). Further analysis of the tumor samples for cell proliferation indicated that knockdown of COUP-TFII reduced cell proliferation as indicated by Ki67 positive cells, and this reduction was abolished by simultaneous repression of MPC1 expression (Figure 5G). All these data support the conclusion that MPC1 plays an essential role in COUP-TFII induction of prostate tumor growth.

## DISCUSSION

COUP-TFII regulates adipogenesis, glucose homeostasis and energy expenditure in normal cells. Unlike normal cells, tumor cells show a distinct metabolic profile with increased glycolysis to generate substrates and energy for proliferation and tumor expansion. Here, we show that COUP-TFII regulates glycolysis to affect prostate cancer cell metabolism. Knockdown of COUP-TFII reduced glucose consumption and lactate production in several prostate cancer cell lines regardless of their differences in the status of AR, PTEN or TP53. We also found that COUP-TFII knockdown reduced NADPH/NADP^+^ ratio in multiple prostate cancer cells (Supplementary Figure S6A and 6B). The reduction of NADPH/NADP^+^ ratio might derive from the fact that reduced glycolysis could lead to reduced material entering into pentose phosphate pathway and thus reduce the NADPH/NADP^+^ ratio. Depletion of COUP-TFII led to the reduction of glycolysis, NADPH/NADP^+^ ratio and ATP levels. All of these suggest that cell growth might be reduced. Indeed, as expected, cell growth is reduced and expression of cell cycle genes are mostly reduced as revealed by mRNA profiling in COUP-TFII knockdown cells [18]. In accordance with the notion that glycolysis contributes to cancer cell metastasis, we found that downregulation of COUP-TFII caused inhibition of cell invasion as shown by the transwell assay. Using an ultra-low attachment culture assay, we also found that downregulation of COUP-TFII caused reduction of the anoikis-resistant growth (data not shown), which is crucial for cancer cells to disseminate, invade and give rise to metastasis.

COUP-TFII regulates a large number of target genes in different cells [20]. In microarray analysis of PC-3 cells, several genes in the glycolysis pathway, including MPC1, are downstream targets of COUP-TFII. We further validated these COUP-TFII regulated genes in prostate cancer cell lines PC-3, LNCaP and CWR22Rv1 using q-PCR. MPC1 was shown to be up-regulated in all these three tested cell lines subsequent to depletion of COUP-TFII. There is a potential COUP-TFII binding site in the MPC1 promoter, and our ChIP assay confirmed binding of COUP-TFII at MPC1 promoter in prostate cancer cells. Mutation of COUP-TFII binding site abrogated COUP-TFII repression of MPC1 promoter driven luciferase activity, suggesting that COUP-TFII directly regulates the transcription of MPC1 by binding to its promoter. However, we didn't find this binding site conserved in the mouse MPC1 promoter, and we did not observe a corresponding COUP-TFII binding peak in mouse embryonic atrial tissues ChIP-Seq dataset (GSE46497), suggesting its species difference.

Bioinformatics analysis, using the online ALGGEN-PROMO program, indicated that the MPC1 promoter contains potential binding sites of E2F, p53, PPAR, SP1 and C/EBP. However, we found that knockdown of neither p53, PPARA, ERG nor SRC-2 could affect MPC1 expression (data not shown). Thus, COUP-TFII is probably the main regulator of the MPC1 expression.

MPC1 and MPC2 were originally known as BRP44L and BRP44, and were recently found to form a complex that controls pyruvate transportation [4, 5, 21]. Although co-expression of both proteins is sufficient to reconstitute pyruvate uptake, the actual transporter complex is about 150kd, which is larger than the two subunits in combination [4]. Thus, it is likely that the transporter is a multimeric complex with other cellular components. John C Schel et al have shown that when overexpressing either MPC1 or MPC2 by itself in colorectal cancer cells, the protein fails to accumulate to a high level, suggesting that these two proteins might need to form a complex to be stable [11]. However, we were able to increase MPC1 protein levels when it was ectopically expressed alone in prostate cancer cells. The reason might be that prostate cancer cells already have elevated expression of endogenous MPC2, which could form functional transporter complexes with the ectopically expressed MPC1. Alternately, there is a cell type specific difference in MPC1/MPC2 stability, since we also found that when MPC1 was knocked down, MPC2 expression remained unchanged in prostate cancer cells, suggesting the complexity of the MPC transporter.

We found that expressing MPC1 in prostate cancer cells reduced cell growth and invasion, which is consistent with the negative correlation between MPC1 expression and patient survival rate, indicating its tumor suppressor role in prostate cancer. Knockdown of MPC1 in prostate cancer cells increased glycolysis and cell invasion, and more importantly abrogated the effect on glycolysis, cell growth and invasion upon COUP-TFII knockdown. Increased glycolysis has long been demonstrated to promote cancer progression through many ways [22, 23]. Recently, repression of MPC1 expression was found not only to increase glycolysis through blocking glucose-derived pyruvate entering into mitochondria, but also to increase the supply of compensatory TCA cycle intermediates from glutamine, amino acids and fatty acids [6, 7]. The TCA cycle and glycolysis provide a synthetic precursor for lipids, proteins and nucleic acids. MPC1 down-regulation mimics a glucose-starved circumstance, which mobilizes or activates usage of different fuel sources to maintain the high levels of precursor pools for cell proliferation, thus promoting cancer progression.

In summary, we have described a role of COUP-TFII in regulating MPC1 expression and glycolysis in prostate cancer. Our data indicate that COUP-TFII regulates prostate cancer metabolism through MPC1 to promote a more aggressive cancer phenotype. Thus, COUP-TFII could serve as a potential target to disrupt prostate cancer cell metabolism and therapeutically benefit prostate cancer patients.

## MATERIALS AND METHODS

### Mammalian cell culture

PC-3, LNCaP, CWR22Rv1 and VCaP cell lines were obtained from ATCC and maintained in RPMI 1640 medium supplemented with 10% FBS (Sigma-Aldrich). LNCaP-abl cell line was maintained in RPMI 1640 medium supplemented with 10% charcoal stripped FBS (Sigma Aldrich). The authenticity of all cell lines was verified in the last 6 months. Cell transfection was performed using Lipofectamine 2000 (Invitrogen) or Dharmacon transfection reagents (Dharmacon), according to the manufacturer's instructions. Lentiviral transductions were performed with lentiviral supernatants generated by cotransfection of 293T cells with vectors VSVG, Δ8.91 and pLV harboring cDNA or vectors pMD2G, PAX2 and pGIPZ harboring shRNA. Stable cells were generated by selection with puromycin (2μg/ml).

### Cell proliferation assay

Cell proliferation assay was performed in a 96-well plate. Proliferation assays were carried out using BrdU Cell Proliferation Chemiluminescent Assay Kit (#5492, Cell Signaling). LNCaP and PC-3 cells were incubated with BrdU for 4 hours and 2 hours respectively. Chemiluminescence was determined using a Multiskan™ FC Microplate Photometer (Thermo Scientific).

### Cell invasion assay

Cell invasion assay was performed using BD BioCoat Matrigel Invasion Chambers (354483, BD Biosciences) according the manufacturer's instruction. PC-3 cells were seeded into the upper chamber at 1×10^4^ cells/well with serum-free medium. Medium with serum was added into the lower chamber. 24 hours later, invaded cells were fixed and stained with 0.1% crystal violet. Photograph was taken, and invaded cells were counted.

### Immunoblotting

Total proteins were extracted from cells following standard protocol. Protein concentration was measured using the BCA protein assay kit (Thermo Fisher Scientific). The primary antibodies used in this study were as follows: COUP-TFII (Cell Signaling), β-actin (Santa Cruz), MPC1 (Sigma-Aldrich), MPC2 (Abgent), P-AMPKa (Thr172) (Cell Signaling), AMPK (Cell Signaling). Horseradish peroxidase (HRP)-conjugated secondary antibodies were purchased from Sigma-Aldrich. Signals were visualized with SuperSignal™ West Dura Extended Duration Substrate (Thermo Fisher Scientific).

### Luciferase assay

293T cells were transfected with indicated vectors including pGL3 vector inserted with MPC1 promoter and pcDNA6 vector inserted with COUP-TFII cDNA. 48 hours after transfection, luciferase activity was determined with ONE-Glo luciferase assay kit (Promega). Forward 5′-GGAAGATCTTTTGGAGACAGGGTCTTGCT-3′ and reverse 5′-CCCAAGCTTAGAGCCAATGACACCCC-3′ primers were used to clone MPC1 promoter (−1194 to 85 region). Forward 5′-ACAGTCCTGTGTTACAGA GAAATTACATTTCCACGTCCCCGCTGGCCG-3′ and reverse 5′-GACGTGGAA ATGTAATTTCTCTGTAACA CAGGACTGTGGGCGCCCTGC-3′ primers were used to clone MPC1 promoter with mutant COUP-TFII binding site. All the primers were synthesized by Sigma-Aldrich.

### Lactate production, glucose consumption, NADPH/NADP^+^ and ATP measurement, glycolysis stress test

Cells were incubated in fresh medium 24 hours after siRNA transfection. 24 hours later, medium were collected for measurement of lactate and glucose using lactate assay kit (Eton Bioscience) and glucose assay kit (Sigma-Aldrich), and cells were subjected to NADPH/NADP^+^ ratio and ATP measurement using NADP/NADPH-Glo™ Assay kit (Promega) and ATP kit (PerkinElmer).

Glycolysis stress test (extracellular acidification rate) was examined using Seahorse instrument XP24 following the manufacturer's instruction. Glucose was used to initiate glycolysis. Oligomycin was used to release glycolytic reserve and reach the maximal glycolysis capacity. 2-DG was used to stop glycolysis. 25,000 PC-3 cells were seeded 24 hours after indicated siRNA transfection for the assay. 10mM glucose, 5μM oligomycin and 20mM 2-DG were used. The results were normalized to total protein concentration.

### q-PCR

Total RNA was extracted from cells using Trizol reagent (15596-018, Life technologies). cDNA was synthesized using Thermo Scientific™ Maxima™ First Strand cDNA Synthesis Kit (FERK1641, Themo Fisher Scientific). Real-time PCR was performed with FastStart Universal SYBR Green Master (4913850001, Roche). The primers are as follows: Forward 5′-TGGCATTGCCGACAGGAT-3′ and reverse 5′-GCTCAGGAGGAGCAATGATCT-3′ for ACTB; forward 5′-CGGGTGGTCGCC TTTATGG-3′ and reverse 5′-ACAGGCATCTGAGGTGAACAG-3′ for COUP-TFII; forward 5′-ATTTGCCTACAAGGTACAGCC-3′ and reverse 5′- AGTCATCTCGTG TTTGATAAGCC-3′ for MPC1.

### Chromatin immunoprecipitation q-PCR assays

Chromatin immunoprecipitation assays were performed using Magna ChIP A/G kit from Millipore according to the manufacturer's protocol. Monoclonal mouse COUP-TFII antibody (R&D), and corresponding control IgG antibodies were used. The quantitative real-time PCR assay was carried out on chromatin samples prepared above. Primer sequences are: primerCBF: 5′-GTCATTGGCTCTGGGAAG-3′ and primerCBR: 5′-CCTTGCTTCGGACATAGT-3′ for COUP-TFII binding site; primerNCF: 5′-AAAGAACAAAGCTGGAGGCA-3′ and primerNCR: 5′-GGGCTC TGTTGTGTTCCA TT-3′ for negative control.

### Xenografts

3×10^6^ PC3 cells mixed with matrigel were subcutaneously injected into the flank of 5 week old male SCID mice. The tumor size was measured 2 weeks later by caliper during the entire experimental process. Tumor volume was calculated by the formula: v=0.5×a×b^2^ (v, the tumor volume; a, the major diameter of the tumor; b, the minor diameter). At the end of the experiment, mice were euthanized, and tumor tissue was removed for further examination.

### Immunochemistry

Immunohistochemistry was done as described previously [19]. Xenograft tumor tissues were fixed with 4% PFA, dehydrated, and embedded in paraffin. The rehydrated tissue slices were treated by heat-mediated antigen retrieval in citrate buffer (Vector Laboratories, Burlingame, CA). The tissues were made permeable with PBS containing 0.2% TritonX-100, then treated with 3% hydrogen peroxide to inactivate endogenous peroxidase. The tissues were washed with PBS containing 0.05% Tween-20, and incubated for 2 h with blocking solution (MOM blocking buffer, Vector Laboratories, CA). Primary Ki67 antibody (550609, BD Biosciences, San Diego, CA, dilution, 1:2000) was incubated overnight at 4°C, and secondary antibodies were incubated for 1h at room temperature. Signals were amplified with ABC kit (Vector Laboratories) and visualized with 3,3′-diaminobenzidine substrate kit (SK-4105, Vector Laboratories). The tissues was further stained with hematoxylin, then dehydrated, and mounted (H5000, Vector Laboratories). Photograph was taken and Ki67 positive cell percentage was calculated.

### Datasets

Taylor dataset (GSE21034) was analyzed for MPC1 expression in patient samples and recurrence-free survival of the patient. It contains 29 adjacent normal, 131 primary and 19 metastasized tumor cases. TCGA dataset was also analyzed for MPC1 expression in patient samples. It contains 498 primary tumor cases and 52 adjacent normal tissues. The Mann-Whitney test was used to identify the MPC1 expression difference between groups.

### GSEA analysis

Gene Set Enrichment Analysis (GSEA) was carried out using the GSEA software from the Broad Institute [24]. Genes regulated by COUP-TFII (4091 genes that were regulated more than 1.2 fold change by COUP-TFII knockdown in PC-3 cells in GSE33182) were ranked by the fold change. 353 genes that down-regulated by at least 1.5 fold following 2-DG treatment in GSE16816 were used as the test set for enrichment analysis. An enrichment score (ES) was calculated in GSEA and the statistical significance of the ES was estimated by the p-value.

### Statistical methods

Data are represented as mean +/− SEM. Statistical analyses were performed using SigmaPlot (San Jose, CA). Unless otherwise noted, data were analyzed by Student's t test and considered significant at p < 0.05.

## SUPPLEMENTARY MATERIAL FIGURES


