# Supplementary material for: MPC1, a key gene in cancer metabolism, is regulated by COUPTFII in human prostate cancer
Source: Oncotarget. 2016 Feb 15;7(12):14673–83. doi: 10.18632/oncotarget.7405 (PMC4924743; doi:10.18632/oncotarget.7405)
Supplement: Supplementary file 1 [file oncotarget-07-14673-s001.pdf]

SUPPLEMENTARY FIGURES

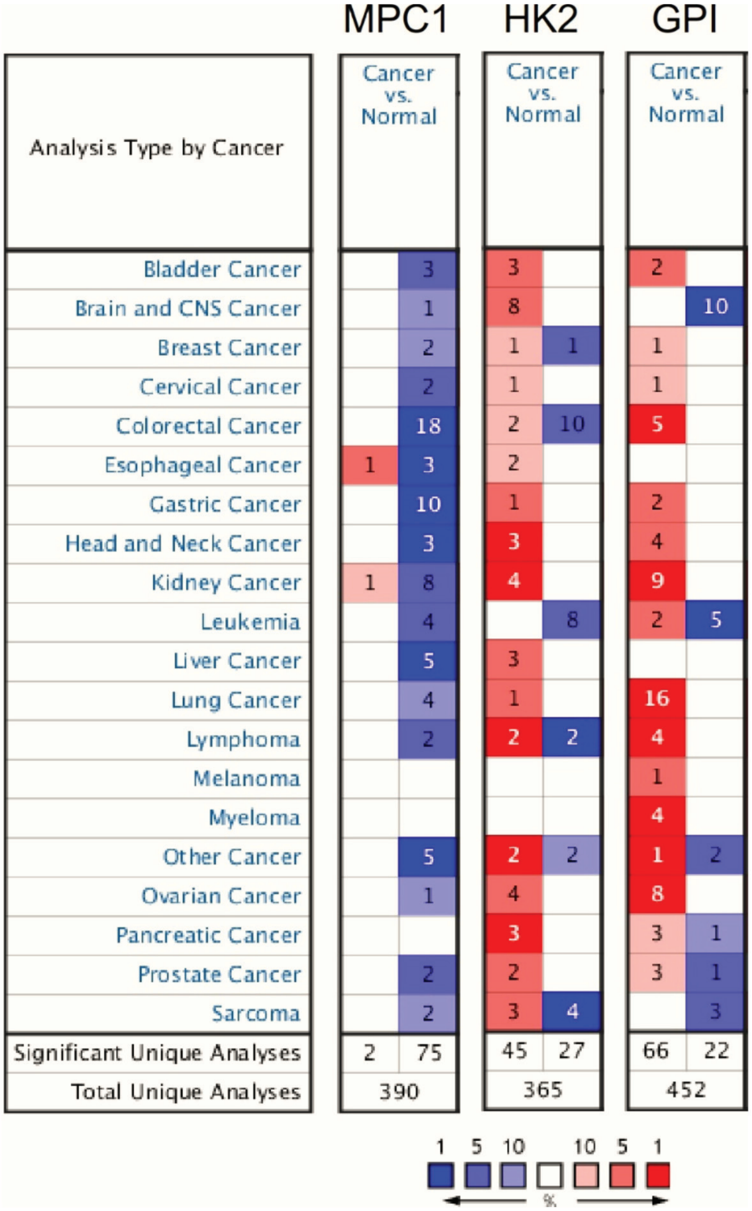

Supplementary Figure S1: Oncomine analysis of MPC1, HK2 and GPI expression profiles across multiple cancer types compared to normal tissue. The number of studies in which upregulation or downregulation was observed is indicated in red or blue boxes, respectively. Color intensity corresponds to the magnitude of expression differences. Threshold (p value) was set to 0.05, threshold (fold change) to 1.5, threshold (gene rank) set to Top 10%; MPC1 queried as BRP44L.

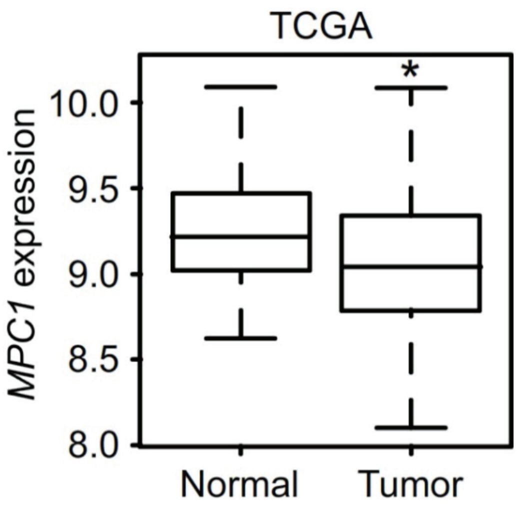

Supplementary Figure S2: MPC1 expression in human prostate cancer samples and control normal adjacent benign prostate from patients in TCGA dataset. *P<0.05; tumor versus normal.

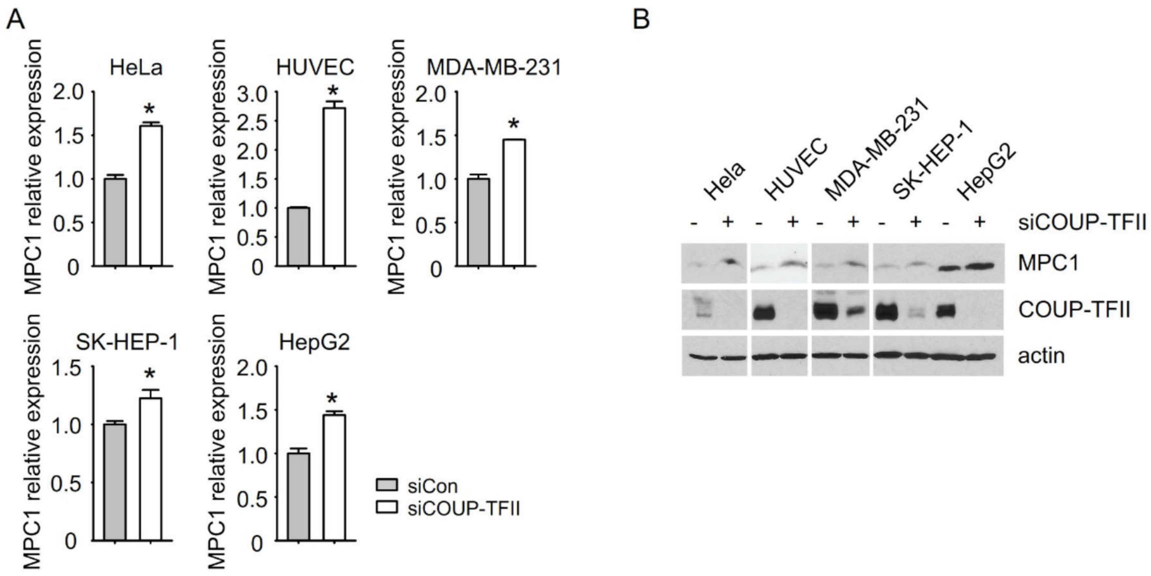

Supplementary Figure S3: MPC1 expression in response to COUP-TFII knockdown in different cell lines. A, B. MPC1 mRNA and protein levels in indicated cell lines were examined by q-PCR and Immunoblotting, respectively at 48 hours after siRNA transfection. *P<0.05.

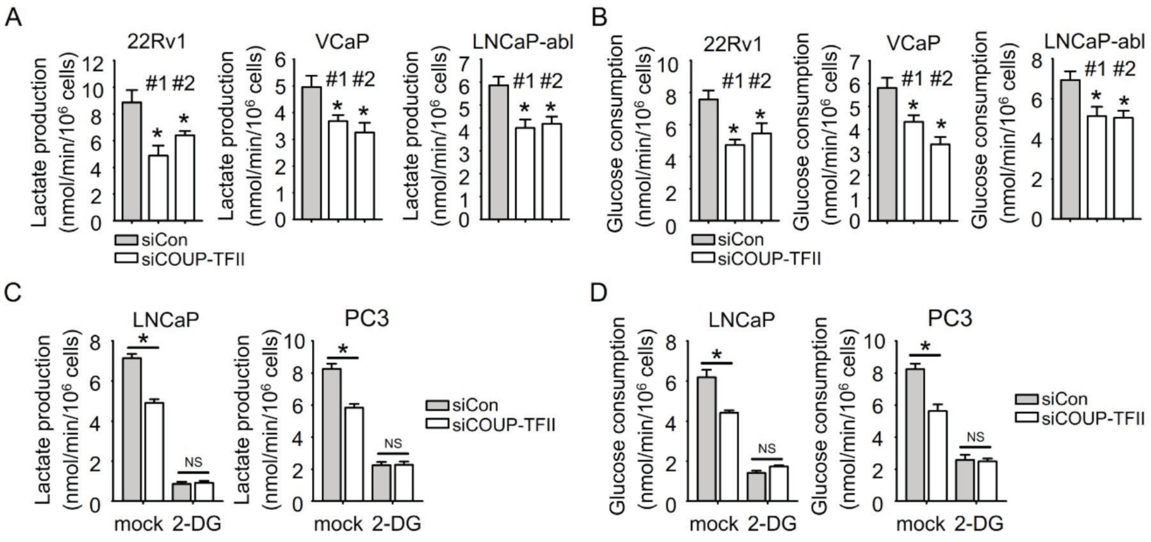

Supplementary Figure S4: COUP-TFII regulates glycolysis in prostate cancer cells. A, B. CWR22Rv1, VCaP and LNCaP-abl cells were transfected with siRNA targeting COUP-TFII. Fresh medium was exchanged at 24 hours after siRNA transfection. After 24 hours, medium was collected for lactate production and glucose consumption assays. *P<0.05. **C, D.** Cells were transfected with siCOUP-TFII, and exchanged for flesh medium containing 2-DG. After 24 hours, medium was collected for lactate production and glucose consumption assays. *P<0.05. NS, difference is not significant.

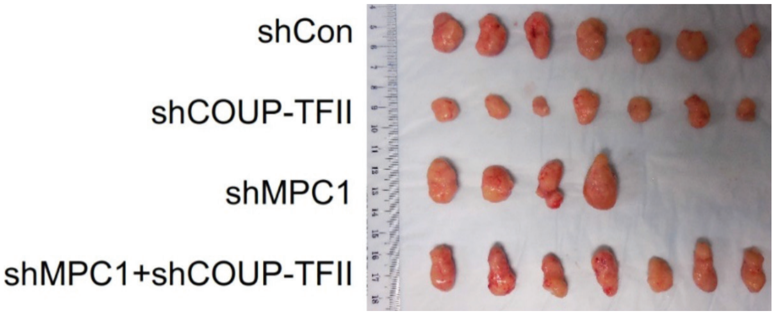

Supplementary Figure S5: Photographs were taken of removed tumor masses corresponding to Figure 5E.

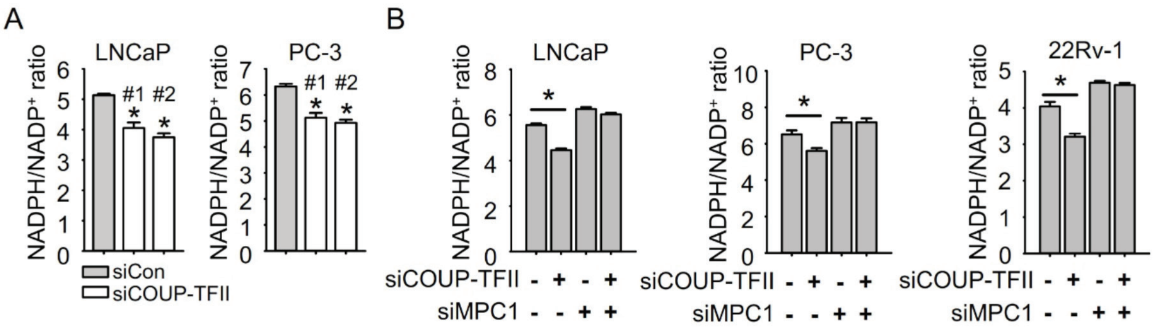

Supplementary Figure S6: A. Two different siRNAs targeting COUP-TFII were transfected into cells. Cells were collected and seeded into 96 well plate at 24 hours after siRNA transfection. NADPH/NADP$^+$ ratio were measured 48 hours later. *P<0.05. **B.** The indicated siRNAs were transfected into cells. Cells were collected and seeded into 96 well plate at 24 hours after siRNA transfection. NADPH/NADP$^+$ ratio were measured 48 hours later. *P<0.05.
